# Clinical decision support tool for diagnosis of COVID-19 in hospitals

**DOI:** 10.1371/journal.pone.0247773

**Published:** 2021-03-11

**Authors:** Claude Saegerman, Allison Gilbert, Anne-Françoise Donneau, Marjorie Gangolf, Anh Nguvet Diep, Cécile Meex, Sébastien Bontems, Marie-Pierre Hayette, Vincent D’Orio, Alexandre Ghuysen

**Affiliations:** 1 Fundamental and Applied Research for Animal and Health (FARAH) Center, University of Liège, Liège, Belgium; 2 Emergency Department, University Hospital Center of Liège, Liège, Belgium; 3 Biostatistics Unit, University of Liège, Liège, Belgium; 4 Public Health Department, University of Liège, Liège, Belgium; 5 Department of Medico-Economic Information, University Hospital Center of Liège, Liège, Belgium; 6 Laboratory of Clinical Microbiology, Center for Interdisciplinary Research on Medicines (CIRM), University Hospital of Liège, Liège, Belgium; International University of Health and Welfare, School of Medicine, JAPAN

## Abstract

**Background:**

The coronavirus infectious disease 19 (COVID-19) pandemic has resulted in significant morbidities, severe acute respiratory failures and subsequently emergency departments’ (EDs) overcrowding in a context of insufficient laboratory testing capacities. The development of decision support tools for real-time clinical diagnosis of COVID-19 is of prime importance to assist patients’ triage and allocate resources for patients at risk.

**Methods and principal findings:**

From March 2 to June 15, 2020, clinical patterns of COVID-19 suspected patients at admission to the EDs of Liège University Hospital, consisting in the recording of eleven symptoms (i.e. dyspnoea, chest pain, rhinorrhoea, sore throat, dry cough, wet cough, diarrhoea, headache, myalgia, fever and anosmia) plus age and gender, were investigated during the first COVID-19 pandemic wave. Indeed, 573 SARS-CoV-2 cases confirmed by qRT-PCR before mid-June 2020, and 1579 suspected cases that were subsequently determined to be qRT-PCR negative for the detection of SARS-CoV-2 were enrolled in this study. Using multivariate binary logistic regression, two most relevant symptoms of COVID-19 were identified in addition of the age of the patient, i.e. fever (odds ratio [OR] = 3.66; 95% CI: 2.97–4.50), dry cough (OR = 1.71; 95% CI: 1.39–2.12), and patients older than 56.5 y (OR = 2.07; 95% CI: 1.67–2.58). Two additional symptoms (chest pain and sore throat) appeared significantly less associated to the confirmed COVID-19 cases with the same OR = 0.73 (95% CI: 0.56–0.94). An overall pondered (by OR) score (OPS) was calculated using all significant predictors. A receiver operating characteristic (ROC) curve was generated and the area under the ROC curve was 0.71 (95% CI: 0.68–0.73) rendering the use of the OPS to discriminate COVID-19 confirmed and unconfirmed patients. The main predictors were confirmed using both sensitivity analysis and classification tree analysis. Interestingly, a significant negative correlation was observed between the OPS and the cycle threshold (Ct values) of the qRT-PCR.

**Conclusion and main significance:**

The proposed approach allows for the use of an interactive and adaptive clinical decision support tool. Using the clinical algorithm developed, a web-based user-interface was created to help nurses and clinicians from EDs with the triage of patients during the second COVID-19 wave.

## Introduction

Infection with the Severe Acute Respiratory Syndrome Coronavirus 2 (SARS-CoV-2) induces the coronavirus infectious disease 19 (COVID-19). The aetiological agent was identified as an unknown β-coronavirus genetically close to SARS-CoV and named SARS-CoV-2 [1]. In confirmed patient, the COVID-19 is characterised by diverse clinical symptoms and mostly, in decreasing order, by history of fever, shortness of breath, and dry cough [2].

Transmission routes of COVID-19 were recently summarised [3]. The main routes are the direct contact [4, 5], and human-to-human transmission by infectious droplets, which are particles > 5–10μm in diameter [6]. Airborne transmission by droplet nuclei, which are generally considered to be particles < 5μm, is also evidenced for SARS-CoV-2 both in experimental [7] and natural [8] conditions. In addition, faecal-oral (e.g. [9, 10]) and ocular [2] transmission routes should also be considered. Due to these modes of transmission, the SARS-CoV-2 spreads very quickly worldwide since its first appearance in China [11].

At the end of 2020, due to the high morbidity of SARS-CoV-2 at world level (≅ 84 millions of people including ≅ 0.65 million in Belgium; https://www.worldometers.info/coronavirus/), the severity of clinical presentation in patients (severe acute respiratory syndrome) and the limited laboratory diagnostic capacities, emergency departments (EDs) have been overwhelmed by the flow of COVID-19 suspected patients in the pandemic context [12]. Moreover, the use of dedicated triage centers is known to offer a management option in disaster situations [13].

Using the two following algorithms (keywords and Boolean operators) in the US National Library of Medicine (PubMed.gov): ((Covid) AND ((clinical presentation) OR (clinical pattern) OR (symptoms) OR (clinical signs)) and ((Covid) AND ((clinical) OR (clinic*) OR (symptom)) AND (decision support tool) AND (hospital)), we found many papers on the clinical presentation of the COVID-19 cases (N = 28,257) (e.g. [2, 14]) but few on clinical decision support tools (N = 46 included 5 review papers) (e.g. [15, 16]). In addition, limited number of patients was involved in most of the studies with some exceptions. However, clinical decision support tools of COVID-19 is crucial to assist patient triage and to allocate resources for patients at risk for severe disease presentation [15].

Our intention is to provide a simple tool available for wider and immediate use. Indeed, the aim of this study is to develop and validate a simple clinical decision support tool of COVID-19 suspected cases in EDs at the hospital in order to translate the result to a hospital IT interface to be directly available at patient’s admission for nurses and clinicians during the second pandemic wave.

## Materials and methods

### Database and study settings

From March 2 to June 15, 2020, a study was performed in the two EDs triage centers close to the University Hospital Center of Liège (CHU). The CHU is composed of two sites (Sart-Tilman and Notre-Dame des Bruyères). In both sites, the triage centers were located near the EDs and were specifically built to manage COVID-19 suspected patients presenting to the EDs. During the study period, all patients directed to the triage centers were eligible to be included. Pediatric patients (age <16 years old) were excluded because the university centers created a specific pediatric ward with particular procedures [12].

During this first COVID-19 wave, a database was created with all information registered in the EDs as the aim was to develop a simple and non-invasive clinical tool. This database was used for the present study. Regarding the symptoms, 11 appealing symptoms (i.e. dyspnoea, chest pain, rhinorrhoea, sore throat, dry cough, wet cough, diarrhoea, headache, myalgia, fever and anosmia) were selected in this study according to the first COVID-19 description [17], the case definition produced by Sciensano, i.e. the Federal Scientific Institute in charge of public health in Belgium [18] and the later description of anosmia [19, 20]. We decide to use only clinical signs plus gender and age in order to develop a simple clinical decision support tool for immediate and real-time use in case of occurrence of a second wave by nurses and clinicians directly at ED’s admission. Those clinical signs were the reported symptoms of patients at admission since the beginning of their disease. Regarding fever, the sign was confirmed positive if the patient reported a body temperature equal or higher to 37.5°C [21].

Binary codification of variables was done: M (male) or F (female) for the gender, less than 56.5 years (coded as 0) and above (coded as 1) for age, and presence (coded as 1) or absence (coded as 0) for all clinical symptoms. It should be noted that, for the age, the variable was numeric only in the classification tree analysis (see below).

We defined three particular periods based on the specific governmental measures applied in Belgium during the first wave:

■ The pre-lockdown or “Early Stage” (March 2 to March 17) which was defined by the finding of the first COVID-19 cases in the Belgian territory.
■ The Lockdown or “Acute Stage” (March 18 to May 3) which was characterized by the announcement of the lockdown in Belgium, implementation of physical distancing, and activation of the hospital emergency plan.
■ The Post-lockdown or “Late Stage” (May 4 to June 15) which represented the end of the full lockdown with a progressive return to usual activities in the healthcare, trading and travelling settings.

### Case definition and real-time reverse transcriptase polymerase chain reaction

A confirmed and unconfirmed COVID-19 case was defined as a person who had a molecularly confirmed and unconfirmed diagnosis of COVID-19 using a quantitative real-time reverse transcriptase polymerase chain reaction (qRT-PCR). During the study period two RT-PCR has been used. The first one to be introduced in March was adapted from the protocol described by Corman et al. [22]. Briefly, viral nucleic acids (RNA) were extracted from clinical samples including mainly nasopharyngeal swabs (300μL) on a Maxwell 48 device using the Maxwell RSC Viral RNA kit (Promega) following a viral inactivation step using Proteinase K according to manufacturer’s instructions. RNA elution occurred in 50μL RNAse free water and 5 μL were used for the RT-PCR. Reverse transcription and RT-PCR were performed on a LC480 thermocycler (Roche) based on Corman et al. protocol [22] for the detection of RdRp and E genes using the Taqman Fast Virus 1-Step Master Mix (Thermo Fisher). The second PCR used is a commercial assay using the cobas® 6800 platform (Roche). For this, 400 μL of nasopharyngeal swabs in a preservative medium (AMIES or UTM) were first incubated at room temperature for 30 minutes with 400 μL of cobas ® PCR Media kit (Roche) for viral inactivation. Samples were then loaded on the cobas® 6800 platform using the cobas® SARS -CoV-2 assay for the detection of the ORF1ab and E genes. Results for both PCRs were expressed as cycle threshold (Ct value, i.e. the number of cycles required for the fluorescent signal to cross the threshold) with limit of positivity being fixed under a Ct 40.

### Scoring system and sensitivity analysis

A scoring system was developed using variables retained in the multivariate binary logistic regression. First, each demographic characteristic or clinical symptom (presence coded as 1) was pondered by its odds ratio (OR) but when an OR was significantly less than one, a reverse codification of this variable was performed (absence recorded as 1 in place of 0) and the ponderation was 1/OR. Finally, all demographic characteristics and clinical symptoms were aggregated as a unique overall pondered score (OPS) by patient (the pondered sum of each variable of interest) (see Eq 2 presented in the results).

A sensitivity analysis was performed to test the robustness of the OPS, consisting of the comparison with the entire data set (as reference) and the same data set removing, one by one, the data from a specific period (i.e. pre-lockdown, lockdown, and post-lockdown) as well as each demographic characteristic or clinical symptoms, or ponderation. In addition, removing two or three variables together were also investigated. After this process, the result of the area under the receiver operating characteristic curve (AUC-ROC; see below) after each removed variable was compared with the AUC-ROC obtained with the reference data set (complete OPS considering all predictors).

### Statistical analysis

#### Basic statistics

Different statistics were used depending of the objective followed. For the representativeness of the patients enrolled in the study, Chi-square test and Pearson correlation coefficient tests were used [23, 24]. The demographic variables between confirmed and unconfirmed COVID-19 cases were compared using a Chi-square test (gender) and a two-sample Wilcoxon rank-sum test (age) [23]. The correlation between demographic characteristics and symptoms retained in the multivariate binary logistic regression and the COVID-19 status of patients (presence-absence data) was assessed using binary Jaccard similarity coefficient [25]. All analyses were performed using Stata SE 14.2 (StataCorp, College Station, Texas, USA). The limit of signification was 0.05.

#### Binary logistic regression and bootstrapped quantile regression

A univariate followed by a multivariate binary logistic regression using backward stepwise approach was used to check the relation between the COVID-19 status of the patients (confirmed or unconfirmed cases) and their demographic characteristics and clinical symptoms [23]. First, the multivariate binary logistic regression included all explanatory variables with a *p*-value ≤ 0.2 as assessed in the univariate binary logistic regression. Secondly, to assess the collinearity, a backward elimination of variables was performed [26]. In this stepwise approach, the non-significant variables (*p*-value > 0.05) were removed starting from the less significant (highest *p*-value). At each step, a likelihood-ratio test comparing the two nested models allowed for the comparison of the simplified with the more complex model. The final model was selected when the likelihood-ratio test highlighted a significant difference between the more complex and the simplified model (p-value < 0.05). Goodness of fit was assessed using the Hosmer–Lemeshow goodness-of-fit test [23].

Due to the difference in the number of observations in function of period (pre-lockdown, lockdown and post-lockdown), the comparison of quartiles of the overall pondered score (see below) was assessed using a bootstrapped quantile regression, an iterative method allowing for the estimation of the parameters of interest based on resampling, previously used in clinical decision-making [24].

All analyses were performed using Stata SE 14.2 (StataCorp, College Station, Texas, USA). The limit of signification was 0.05.

#### Receiver operating characteristic curve

A ROC curve (probability curve) was plotted with true positive results (Y-Axis) against the false positive results (X-Axis). The AUC-ROC is the performance measurement for the classification of the OPS (see above) at various thresholds settings. The higher the AUC-ROC, the better the OPS is able to distinguish between confirmed and unconfirmed COVID-19 cases (i.e. measurement of the separation of the two sub-populations). In addition, the Youden’s index “J” is frequently used in conjunction with the ROC curve analysis to estimate the best cut-off [23], with:

Youden’sindex=sensitivity+specificity−1 [Eq 1]

The value of AUC-ROC ranges from 0 to 1 (inclusive). A zero value is observed when a diagnostic test gives the same proportion of positive results for groups confirmed or unconfirmed COVID-19 cases. A value of 1 indicates that there are no false positives or false negatives, i.e. the test is perfect. In a ROC curve the calculation of the Youden’s index in all points allows to determine the best cut-off of the test (i.e. optimal Youden’s index) [23].

#### Classification and regression tree analysis

A classification and regression tree (CART) analysis was conducted on the data set. The dependent variable is COVID-19 status (confirmed versus unconfirmed cases). The independent variables depended on the objective followed. For the classification tree analysis (CTA), the independent variables were the demographic characteristics and clinical symptoms, which were significant in the univariate binary logistic regression (see above). For the regression tree analysis (RTA), the independent variable was the OPS. A CART analysis is a non-linear and non-parametric model that is fitted by binary recursive partitioning of multidimensional covariate space [27–30]. Using Salford Predictive Modeler (SPM) 8.3.2. (Minitab LLC, Stade College, PA, USA) [31], the analysis successively splits the data set into increasingly homogeneous subsets. The Gini index was used as the splitting method, and 10-fold cross-validation was used to test the predictive capacity of the obtained trees [27]. CART performs cross-validation by growing maximal trees on subsets of data, then calculating error rates based on unused portions of the data set. To accomplish this, CART divides the data set into 10 randomly selected and roughly equal parts, with each ‘part’ containing a similar distribution of the data from the populations of interest (i.e. COVID-19 confirmed versus unconfirmed cases). CART then uses the first nine parts of the data, constructs the largest possible tree and uses the remaining 1/10 of the data to obtain initial estimates of the error rate of the selected subtree. The process is repeated using different combinations of the remaining nine subsets of data and a different 1/10 data subset to test the resulting tree. This process is repeated until each 1/10 subset of the data has been used to test a tree that has been grown using a 9/10 data subset. The results of the 10 mini-tests are then combined to calculate error rates for trees of each possible size. These error rates are applied to prune the tree grown using the entire data set. The consequence of this complex process is a set of reliable estimates of the independent predictive accuracy of the tree. For each node in a CART analysis-generated tree, the ‘primary splitter’ is the variable that best splits the node, maximizing the purity of the resulting nodes.

## Results

### Description of the studied patients

**Fig 1:**
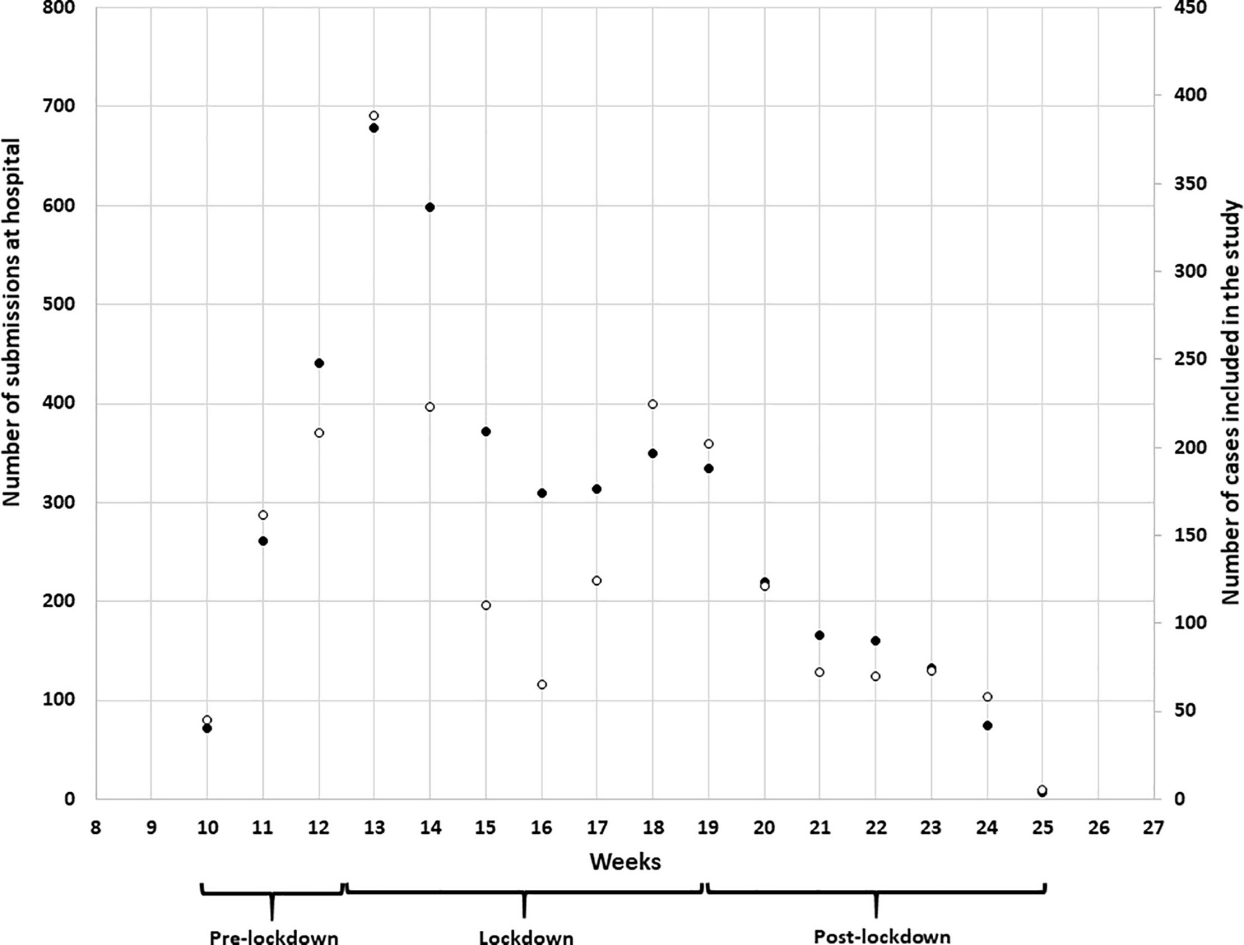
Number of patients suspected to be infected with SARS-CoV-2 directed to the triage centers located close to the emerging departments of the Liège University Hospital (N = 4,489) in function of weeks as well as those that were tested by qRT-PCR and included in the present study (N = 2,152). Patients suspected to be infected with SARS-CoV-2 (black circles) and patients tested by qRT-PCR and included in the study (white circles).

Among 4,489 patients suspected to be infected with the SARS-CoV-2 (called as ALL-data set), 2,152 patients (47.9%) were enrolled in the clinical study due to the presence of full clinical records (without missing values) and a qRT-PCR test result (**Fig 1**). This number included 573 SARS-CoV-2 cases (26.6%) confirmed by qRT-PCR before mid-June 2020, and 1579 suspected cases (73.4%) that were subsequently determined to be qRT-PCR negative for the detection of SARS-Cov-2 (called as STUDY-data set). The weekly number of patients included in the clinical study was highly correlated to the whole data set of patients suspected to be infected with the SARS-CoV-2 (Pearson correlation coefficient = 0.895 with *p*-value < 0.0001). The characteristics of the study population are summarized in **Table 1**.

**Table 1:** Demographic characteristics and clinical symptoms of the study population of patients (N = 2,152).

Variable	COVID-19 qRT-PCR test result	
	Positive	Negative
Demographic characteristics		
Age,		
mean (SD), y	58 (19.5)	52 (19.1)
median (P25-P75), y	59 (40–73)	51 (35–66)
Female sex, No. (%)	294 (51.3)	913 (57.8)
Clinical symptoms (presence)		
Dyspnoea, No. (%)	293 (51.1)	729 (46.2)
Chest pain, No. (%)	109 (19.0)	401 (25.4)
Rhinorrhoea, No. (%)	176 (30.7)	521 (33.0)
Sore throat, No. (%)	117 (20.4)	420 (26.6)
Dry cough, No. (%)	296 (51.7)	639 (40.5)
Wet cough, No. (%)	78 (13.6)	210 (13.3)
Diarrhoea, No. (%)	125 (21.8)	318 (20.1)
Headache, No. (%)	234 (40.8)	614 (38.9)
Myalgia, No. (%)	230 (40.1)	570 (36.1)
Fever, No. (%)	368 (64.2)	550 (34.8)
Anosmia, No. (%)	7 (1.2)	35 (2.2)
Period of admission		
Pre-lockdown (early stage), No. (%)	56 (9.8)	224 (14.2)
Lockdown (acute stage), No. (%)	498 (86.9)	782 (49.5)
Post-lockdown (late stage), No. (%)	19 (3.3)	573 (36.3)

SD, standard deviation; No, number; %, percentage; and qRT-PCR, real-time reverse transcription polymerase chain reaction; P25, percentile 25; P75, percentile 75.

The representativeness of the patients enrolled in the study and tested for SARS-CoV-2 using qRT-PCR in comparison with all patients suspected to be infected with SARS-CoV-2 directed to the triage centers was indirectly assessed using two demographic characteristics (i.e. gender and age). There was no significant difference as regard to the gender between the sampling study and the sampling frame (Chi2 _(1 df; α = 0.05)_ = 0.98; *p*-value = 0.0004). The median age was significantly different (Chi2 _(4 df; α = 0.05)_ = 17.88; *p*-value = 0.001) with only a difference of 2 years. However, the number of patients in the STUDY-data set and ALL-data set, in the five classes of age recommended by United Nations (1982) [32], i.e. 1–24 y, 25–54 y, 55–74 y, 75–84 y and ≥ 85 y, was highly correlated (Pearson correlation coefficient = 0.99; p-value = 0.0008).

### Time between the appearance of the first clinical signs and the arrival at the hospital

No statistical difference was observed between patients tested positive (median = 4 days; interquartile [IQR] = 6 days) and patients tested negative (median = 3 days; IQR = 6.25 days) for the SARS-CoV-2 regarding the time between the appearance of the first clinical symptoms and the arrival at the hospital (Two-sample Wilcoxon rank-sum test; *p*-value > 0.05).

### Comparison of demographic characteristics and clinical symptoms in patients SARS-CoV-2 confirmed and unconfirmed by qRT-PCR

Confirmed COVID-19 cases (median = 59 y; IQR = 33 y) were found to be statistically older than the unconfirmed cases (median = 51 y; IQR = 31 y) (Two-sample Wilcoxon rank-sum test; *p*-value <0.0001). Using a classification tree analysis, the best cut-off to discriminate the two groups according to age was 56.5 years. Indeed, a recodification was performed with patients less than 56.5 y as 0 and with patient more than 56.5 y as 1 (binary transformation).

In addition, there were statistically less females within the COVID-19 confirmed cases (51.3%) in comparison with the unconfirmed COVID-19 cases (57.8%) (Pearson Chi2 _(1 degree of freedom and α = 0.05)_ = 7.24; *p*-value = 0.007).

Using both univariate (**Table 2**) and multivariate (**Table 3**) binary logistic regression, some demographic characteristics and clinical symptoms were found to be statistically frequently observed in COVID-19 confirmed cases compared to the unconfirmed cases.

**Table 2:** Odds ratio with 95% confidence interval (CI) derived from univariate binary logistic regression, for two demographic characteristics (age and gender) and eleven symptoms in 573 confirmed and 1579 unconfirmed COVID-19 patients.

Variable	Presence	OR	(95% CI)	*p*-value	
Demographic characteristics					
Age	< 56.5 y	Reference	-	-	
	≥ 56.5 y	1.79	(1.48–2.17)	<0.001	*
Gender	Male	Reference	-	-	
	Female	0.77	(0.63–0.93)	0.007	^#^
Symptoms					
Dyspnoea	No	Reference	-	-	
	Yes	1.22	(1.01–1.48)	0.042	*
Chest pain	No	Reference	-	-	
	Yes	0.69	(0.54–0.875)	0.002	^#^
Rhinorrhoea	No	Reference	-	-	
	Yes	0.90	(0.73–1.11)	0.32	
Sore throat	No	Reference	-	-	
	Yes	0.71	(0.56–0.89)	0.004	^#^
Dry cough	No	Reference	-	-	
	Yes	1.57	(1.29–1.91)	<0.001	*
Wet cough	No	Reference	-	-	
	Yes	1.03	(0.78–1.36)	0.85	
Diarrhoea	No	Reference	-	-	
	Yes	1.11	(0.88–1.40)	0.40	
Headache	No	Reference	-	-	
	Yes	1.08	(0.89–1.32)	0.41	
Myalgia	No	Reference	-	-	
	Yes	1.19	(0.98–1.44)	0.09	
Fever	No	Reference	-	-	
	Yes	3.36	(2.75–4.10)	<0.001	*
Anosmia	No	Reference	-	-	
	Yes	0.55	(0.24–1.24)	0.15	

OR, odds ratio; CI, confidence interval

* risk explanatory variable

^#^ protective explanatory variable.

**Table 3:** Odds ratio with 95% Confidence Interval (CI) derived from multivariate binary logistic regression, calculated for two demographic characteristics (age and gender) and symptoms which were found to be significant at the univariate level, when considering p-value < 0.2, in 573 confirmed and 1579 unconfirmed COVID-19 patients.

Variable	Presence	OR	(95% CI)	*p*-value	
Demographic characteristics					
Age	< 56.5 y	Reference	-	-	
	≥ 56.5 y	2.07	(1.67–2.58)	<0.001	*
Symptoms					
Dyspnoea	No	Reference	-	-	
	Yes	1.23	(0.99–1.52)	0.06	
Chest pain	No	Reference	-	-	
	Yes	0.73	(0.56–0.94)	0.017	^#^
Sore throat	No	Reference	-	-	
	Yes	0.73	(0.56–0.94)	0.015	^#^
Dry cough	No	Reference	-	-	
	Yes	1.71	(1.39–2.12)	<0.001	*
Fever	No	Reference	-	-	
	Yes	3.66	(2.97–4.50)	<0.001	*

OR, odds ratio; CI, confidence interval

* risk explanatory variable

^#^ protective explanatory variable.

The same risk or protective explanatory variables were identified using both univariate and multivariate binary logistic regression, except for dyspnoea (*p*-value was close to the limit of significance for the final regression modelling). The Hosmer–Lemeshow goodness-of-fit test showed that the model adequately fit the data (Hosmer-Lemeshow Chi2 (8 df) = 7.67 with p-value = 0.47).

### Correlation matrix between demographic characteristics and symptoms retained in the multivariate binary logistic regression and the COVID-19 status of patients

In order to visualize the possible association between symptoms and the COVID-19 status of patients, a matrix of binary Jaccard similarity coefficients was calculated (**Table 4**). The COVID-19 status was more associated with, in decrease order, fever, age, dry cough and dyspnoea. Age was more associated with dyspnoea. Fever was more associated with dry cough and dyspnoea; dry cough with dyspnoea and sore throat, and dyspnoea with chest pain.

**Table 4:** Matrix of binary Jaccard similarity coefficients between clinical signs and the COVID-19 status.

	COVID-19 status	Age	Fever	Dry cough	Dyspnoea	Chest pain	Sore throat
COVID-19 status	1.000						
Age	0.260	1.000					
Fever	0.328	0.232	1.000				
Dry cough	0.244	0.213	0.320	1.000			
Dyspnoea	0.225	0.379	0.265	0.315	1.000		
Chest pain	0.112	0.144	0.157	0.213	0.306	1.000	
Sore throat	0.118	0.091	0.205	0.283	0.189	0.205	1.000

Code of colour for the coefficients in function of the increasing importance of the binary similarity (green to red).

### Overall pondered clinical score and area under the receiver operating characteristic curve

A pondered score was calculated using significant demographic variables (i.e. age) and four clinical characteristics of patients as explanatory variables (see **Table 3**). Finally, all characteristics were aggregated as a unique overall pondered score (OPS) by patient (the pondered sum of each variable of interest) using the following formula:

$$OPS=\left[\left(Presence_{age>56.5y}=1\right)*\left(OR_{age}\right)\right]\;{+}_{}\left[\left(Absence_{chest\;pain}=1\right)*\left(1/OR_{chest\;pain}\right)\right]\;{+}_{}\left[\left(Absence_{sore\;throat}=1\right)*\left(1/OR_{sore\;throat}\right)\right]+\left[\left(Presence_{dry\;cough}=1\right)*\left(OR_{dry\;cough}\right)\right]+\left[\left(Presence_{fever}=1\right)*\left(OR_{fecer}\right)\right]$$ [Eq 2]

With: OPS, overall pondered score; OR, odds ratio presented in **Table 3**. With this formula, the minimum and the maximum theoretical values of the OPS are 0 and 10.18.

**Fig 2:**
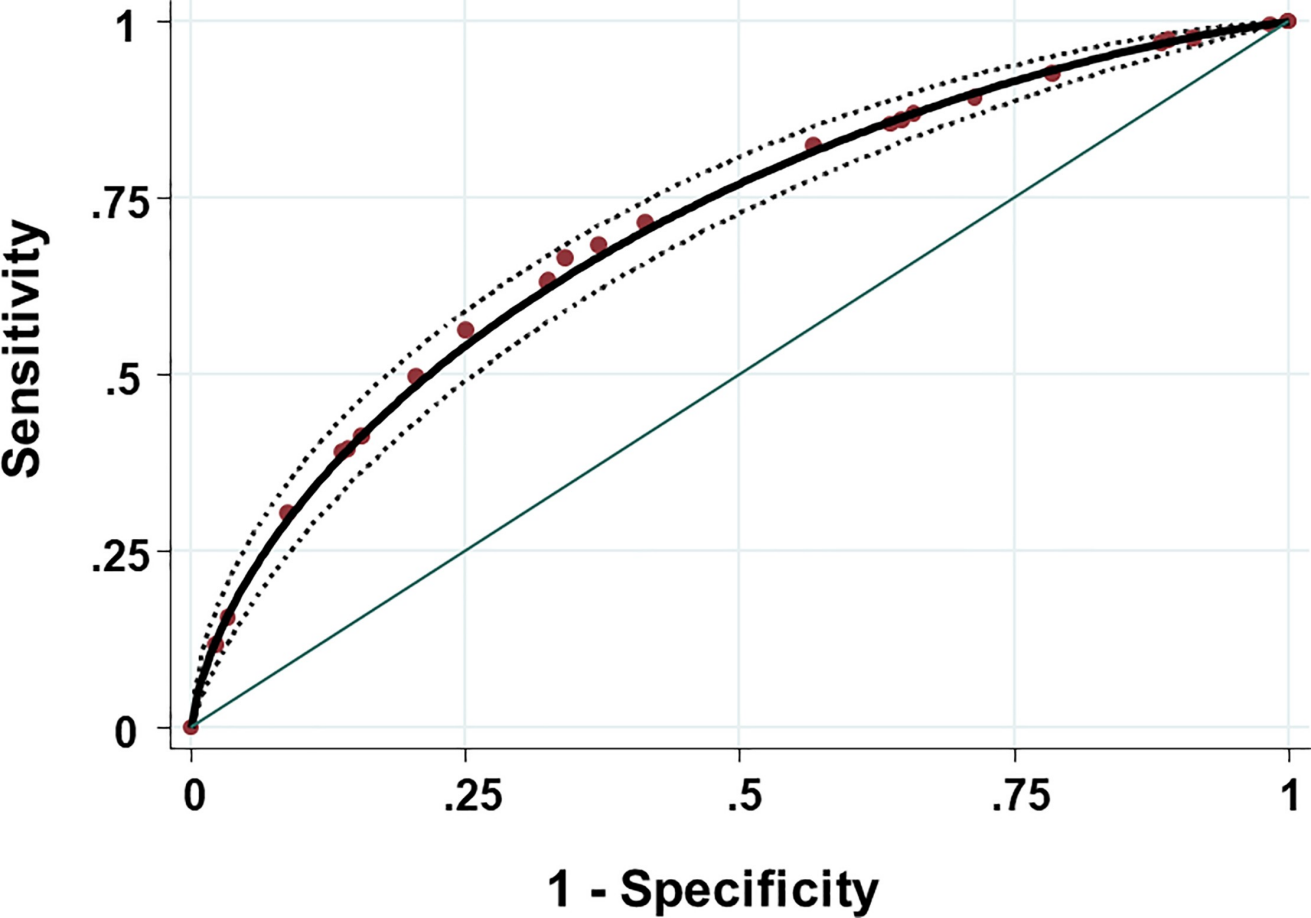
Receiver operating characteristic curve of the overall pondered score of COVID-19. Points are the observed values; the solid curve in black and its 95% confidence interval (broken curves in black) was fitted according to a binormal distribution. Area under curve = 0.71 (95% CI: 0.68–0.73) with standard error = 0.012.

The clinical diagnostic discriminatory power was assessed by calculating the AUC-ROC (**Fig 2**). The AUC-ROC was 0.71 (95% CI: 0.69–0.73) with standard error = 0.013. Using both the Youden index (i.e. 0.32) and a regression tree analysis, the best cut-off to discriminate the two sub-groups (positive and negative patients to SARS-CoV-2) was OPS = 5.07. Applying this cut-off, the sensitivity and the specificity were 66.5% (95% CI: 62.5–70.4) and 65.9% (95% CI: 63.5–68.2), respectively.

**Fig 3:**
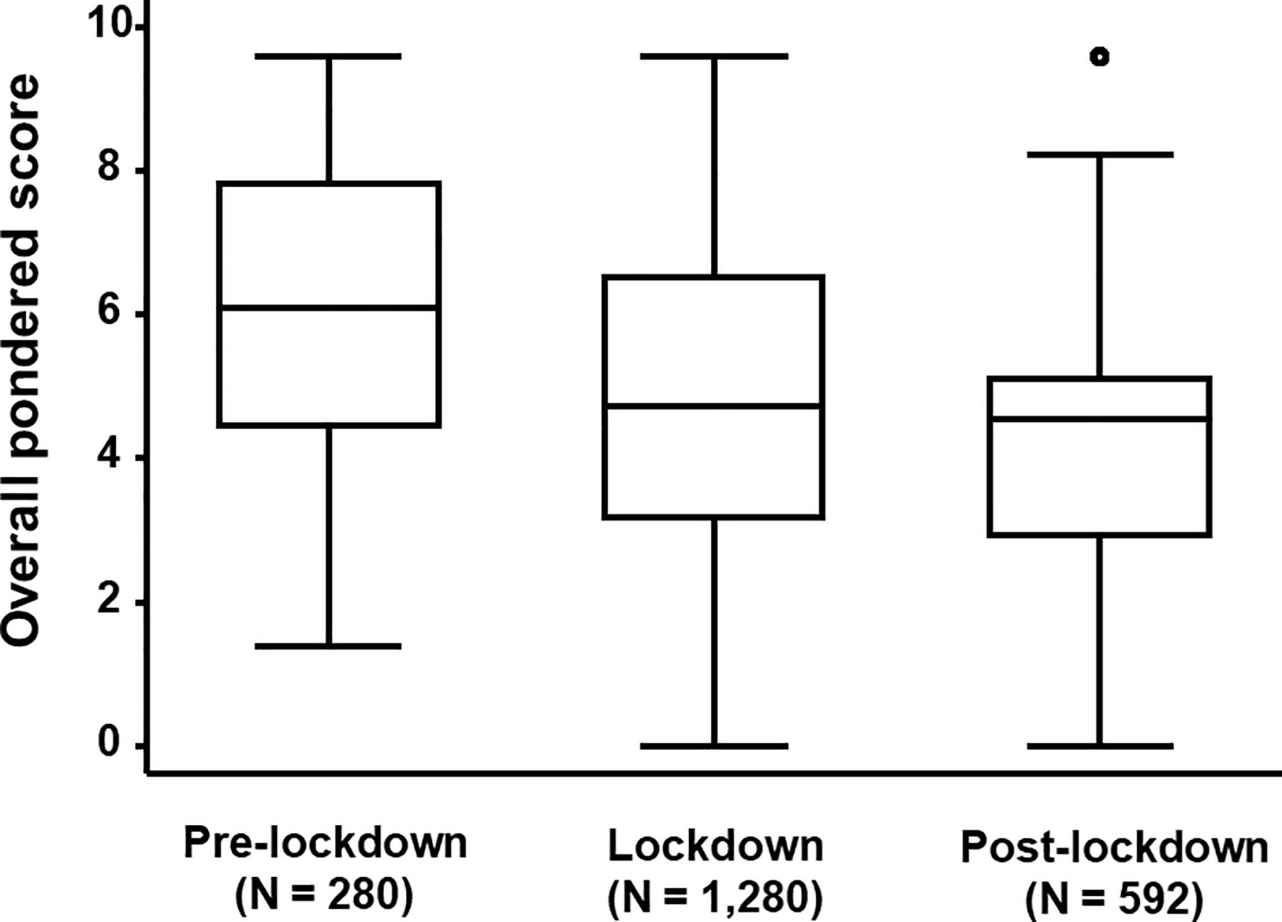
The evolving of the Overall Pondered Score (OPS) in function of the period of the first COVID-19 pandemic wave.

The evolving of the OPS in function of the period, i.e. pre-lockdown (N = 280 observations), lockdown (N = 1280 observations) and post-lockdown (N = 592 observations) was analysed (**Fig 3**). Because of the difference in the number of observations in function of period, we opted to test the quartiles, using a bootstrapped (N = 200) quantile regression. A significant decrease of the median in function of the period was observed (*p*-value < 0.001). The median of the pre-lockdown, lockdown and post-lockdown period was 6.11 (IQR: 3.36), 4.73 (IQR: 3.36) and 4.54 (IQR: 2.19), respectively.

The probability of confirmed and unconfirmed COVID-19 cases in function of the result of the OPS is depicted in **Table 5**.

**Table 5:** The probability for a patient to be confirmed and unconfirmed COVID-19 cases in function of the result of the Overall Pondered Score (OPS).

OPS	COVID-19		Total	Probability		Proposed level
	Confirmed	Unconfirmed		Confirmed	Unconfirmed	
**[0–1]**	2	26	28	**0.07**	**0.93**	**Level 1**
**[1–2]**	15	156	171	**0.09**	**0.91**	
**[2–3]**	25	158	183	**0.14**	**0.86**	
**[3–4]**	41	232	273	**0.15**	**0.85**	
**[4–5]**	109	468	577	**0.19**	**0.81**	
**[5–6]**	19	26	45	**0.42**	**0.58**	**Level 2**
**[6–7]**	139	296	435	**0.32**	**0.68**	
**[7–8]**	134	164	298	**0.45**	**0.55**	
**[8–9]**	22	18	40	**0.55**	**0.45**	**Level 3**
**[9–10]**	67	35	102	**0.66**	**0.34**	
**Total**	**573**	**1579**	**2152**			

For the probability, the scale of colour is related to the increasing of its value (green to red). Proposed level: Level 1, high probability for a patient to become unconfirmed by qRT-PCR; Level 2, intermediate level; and Level 3, high probability for a patient to become confirmed by qRT-PCR.

In addition, for SARS-CoV-2 cases confirmed by qRT-PCR, a significant negative correlation between the Ct values of the qRT-PCR and the OPS was observed (Pearson correlation coefficient = -0.14 with *p*-value = 0.001).

### Classification tree analysis

**Fig 4:**
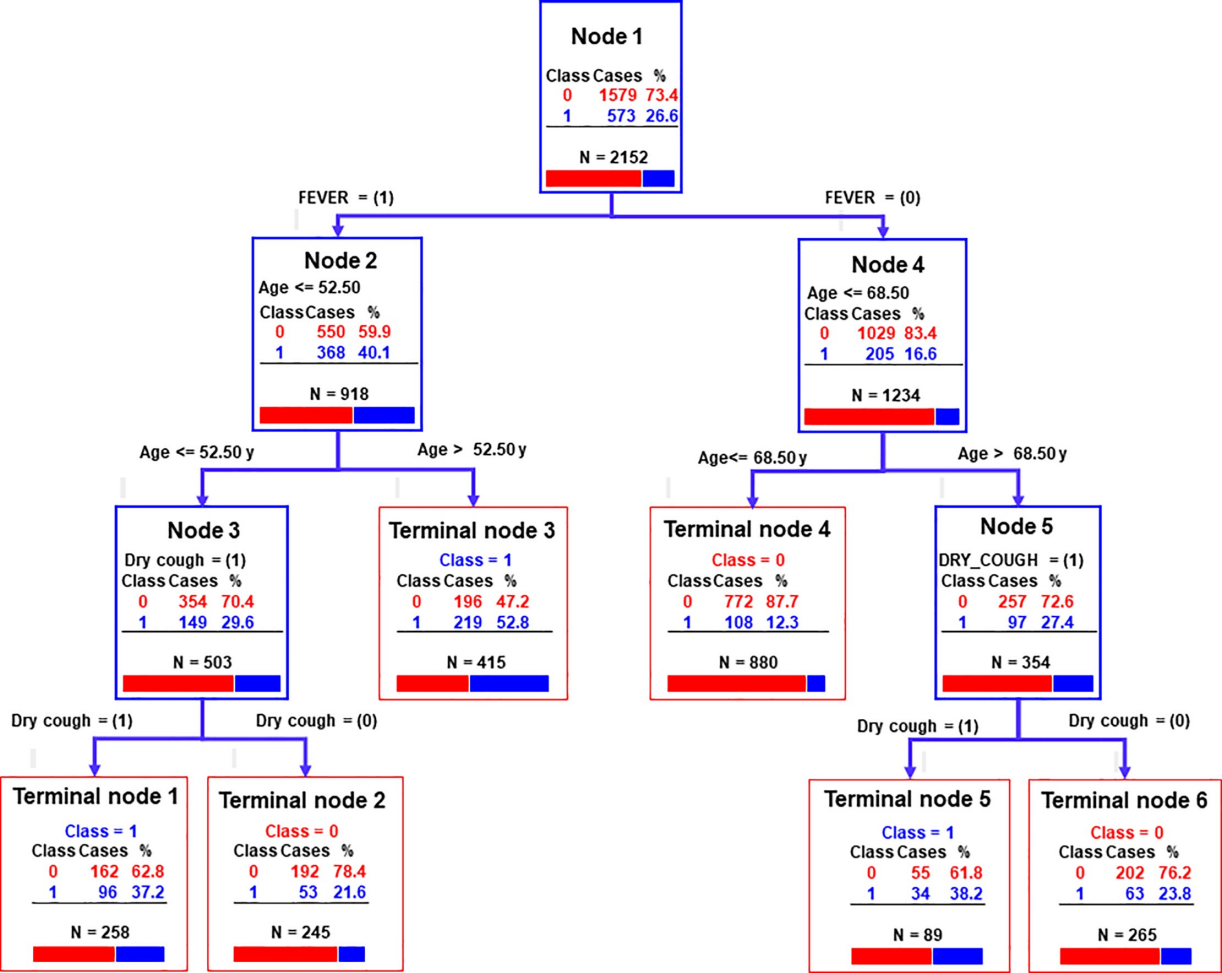
Classification decision tree for clinically suspected COVID-19 cases at the Liège University Hospital (N = 2,152). (1) and (0), presence and absence of the symptom; Class, in blue or red, the number of confirmed or unconfirmed patients to SARS-CoV-2, respectively.

Using the two significant demographic characteristics (note that here, age was expressed in years) and five symptoms of patient determined by the use of the univariate binary logistic regression (see **Table 2**), a classification tree analysis was built (**Fig 4**). The relative importance (RI) of the demographics and five symptoms of the patients as follows, in decreasing order (scale from 0 to 100): fever (RI: 100), age in years (RI: 81.3), dry cough (RI: 35.9), gender (RI: 15.4), dyspnoea (RI: 11), sore throat (10.6) and chest pain (RI: 7.1). The AUC-ROC for the learning data set and the testing data set was 0.71 and 0.69 respectively. The sensitivity of the clinical decision tree for the learning data set and for the testing data set was 62.0% (95% CI: 57.8–65.9) and 60.4% (95% CI: 56.2–64.4). The specificity of the clinical decision tree for the learning data set and for the testing data set was 73.8% (95% CI: 71.6–76.0) and 70.0% (95% CI: 67.7–72.3).

### Sensitivity analysis

A sensitivity analysis was performed to assess the robustness of Eq 2, consisting of the comparison with the entire data set (as reference) and the same data set removing, one by one, the data from a specific period (i.e. pre-lockdown, lockdown, and post-lockdown) as well as each demographic characteristic (age) or symptom, or ponderation. To monitor the effect, the four resulting AUC-ROC were compared (**Table 6**). In addition, the effect of removing two or three variables together were also investigated. Considering the respective standard error, no important difference of the AUC-ROC was observed in function of the data set used except for fever alone, both age and fever, both fever and dry cough, and age with fever and dry cough together (distinct range of 95% CI). Note that the standard error is higher when data gained in the lockdown were removed.

**Table 6:** Area under the receiver operating characteristic curve (AUC-ROC) in function of the data set used.

Data set	AUC-ROC (95% CI)	Standard error
All demographic characteristic and relevant symptoms included (as a reference) = ALL	0.71 (0.68–0.73)	0.013
ALL periods minus pre-lockdown	0.72 (0.69–0.75)	0.013
ALL periods minus lockdown	0.68 (0.61–0.74)	0.033
ALL periods minus post-lockdown	0.69 (0.66–0.71)	0.014
ALL minus age	0.69 (0.66–0.71)	0.013
ALL minus chest pain	0.70 (0.68–0.73)	0.013
ALL minus sore throat	0.70 (0.68–0.73)	0.013
ALL minus dry cough	0.70 (0.67–0.72)	0.013
ALL minus fever	0.62 (0.60–0.65)*	0.014
ALL minus fever & dry cough	0.60 (0.57–0.63)*	0.015
ALL minus age & fever	0.60 (0.57–0.63)*	0.015
ALL minus age & dry cough	0.68 (0.66–0.71)	0.014
ALL minus age & chest pain	0.69 (0.66–0.71)	0.013
ALL minus age & sore throat	0.68 (0.66–0.71)	0.013
ALL minus chest pain & sore throat	0.70 (0.67–0.73)	0.013
ALL minus age & fever & dry cough	0.58 (0.54–0.62)*	0.021
ALL minus age & chest pain & sore throat	0.67 (0.64–0.69)	0.013
ALL minus ponderation	0.69 (0.66–0.72)	0.013

All periods represent the pre-lockdown, the lockdown and the post-lockdown together; CI, confidence interval

* Significant difference regarding the reference data set (absence of overlapping between 95% CI).

## Discussion

From March 2 to June 15, 2020, clinical patterns of COVID-19 suspected patients at admission to the EDs of Liège University Hospital, consisting in the recording of 11 symptoms (i.e. dyspnoea, chest pain, rhinorrhoea, sore throat, dry cough, wet cough, diarrhoea, headache, myalgia, fever and anosmia) plus age and gender, were investigated during the first COVID-19 pandemic wave.

No notable statistical difference was evidenced between patients enrolled in this study (i.e. with full clinical records and tested for SARS-CoV-2 by qRT-PCR) and patients admitted to the EDs of the hospital, allowing the possible generalisation of the results of the study to all admitted patients.

The main results of the study indicate the usefulness of a combination and weighting of key demographic characteristics and symptoms as clinical decision support tool.

Concerning the demographic characteristics, in this study, female patients were less at risk to be confirmed as COVID-19 positive, but only in the univariate binary logistic regression. This finding can be related to the fact that male was previously identified as a main predictor but to intensive care unit [33]. However, that this symptom was not retained in the final multivariate binary logistic regression of this study is perhaps related to the fact that no targeting of intensive care was done. Confirmed cases of COVID-19 were also older than the unconfirmed cases. This finding is in accordance with previous studies (e.g. [34, 35]).

Symptoms that are significantly more (dyspnoea, dry cough and fever) or less (chest pain and sore throat) prevalent in patients confirmed as COVID-19 by qRT-PCR are the same both in univariate and multivariate binary logistic regression except for dyspnoea for which the *p*-value was 0.06 in the final multivariate binary logistic regression (close to the limit of significance). Interestingly, in a large cohort of patients (mostly laboratory-confirmed for SARS-CoV-2 infection), the International Severe Acute Respiratory and Emerging Infections Consortium [36] ranked fever (presence in 67% of confirmed cases), shortness of breath ≅ dyspnoea (presence in 64% of confirmed cases) and dry cough (presence in 43% of confirmed cases) as the top of symptoms more frequent, and chest pain and sore throat with low frequency (< 15%) [2]. While, the ISARIC database contains mostly laboratory confirmed COVID-19 cases, comparison with unconfirmed COVID-19 cases was not available.

Using one demographic characteristic (age) and four clinical symptoms, i.e. two most frequently found in confirmed SARS-CoV-2 cases and two most frequently found in unconfirmed SARS-CoV-2 cases, respectively, the AUC-ROC of the OPS of COVID-19 was 0.71. This value means that our model can predict the presence of confirmed SARS-CoV-2 by qRT-PCR with an accuracy of 71%. According to the Swets J.A. (1988) scale [37], this model was estimated as useful. This simple model should be translated into the hospital’s IT interface to be directly available as a clinical decision-support tool at ED’s admission for nurses and clinicians during the occurrence of a second wave. Data registered with IT interface should be included in a clinical database allowing real-time analysis and permits direct subsequent validation and refining of the tool (i.e. Eq 2) to capture possible evolution of the clinical patterns over time. At the time of the revision of the paper, a preliminary check on a limited data set of the second wave (N = 261 first patients), claims for an external validity of the developed clinical decision tree, as the AUC-ROC = 0.65 (Saegerman C., personal communication). In order to flexibly render the tool, we recommend the inclusion of at least two main demographic characteristics easy to collect (age and gender) and a selection of about ten symptoms referred by the ISARIC [36] but with some first ranked (e.g. fever, dry cough and dyspnoea) and some less ranked (e.g. chest pain, sore throat). In the past, other powerful clinical decision support tools were developed and found that the combination of clinical signs with different frequencies (high and low) are valuable, especially for emerging diseases (e.g. [24, 28, 38–40]).

Concerning fever, results seem to differ from study to study. Memni et al [41] found fever significant in their US cohort but not in their UK cohort. Our study considered fever as a significant variable. The difference between the models could be due to the recording of symptoms and/or the effect of media reports. In our study, the symptoms were recorded at the hospital (the patient was examined by a medical doctor who has carried out an anamnesis in the hospital environment) whereas in the study of Memni et al. [41], the symptoms were self-reported by the patient. The perception of fever by the patients and the assessment of fever by a practitioner maybe factors that lead to differences in studies results. More generally, the difference between studies should be due to convenience sampling and method of data collection. Therefore, such a comparison between studies needs a thorough analysis of the design and the protocol used. In addition, due to health emergency needs, most of past and current studies are not interventional.

Concerning anosmia (loss of smell and taste), its description in COVID-19 patients [31, 32] was later in the first wave. In addition, in Memni et al. [41], this was considered as a potential symptom of COVID-19 but also increasing following media reports. Indeed, due to the period of the present study, a possible bias could exist. A follow-up of this symptom is needed in future studies.

Some recent modelling including many variables (demographic, clinical, laboratory, and exposure-risk variables ascertainable at presentation) was performed to develop algorithms for estimating the risk of COVID-19 (e.g. [34]), with a higher AUC-ROC observed for some models. However, the advantage of the proposed model in this study is due to the real-time capture of demographic characteristics and symptoms presented at the admission and these should be used directly to appropriately manage the triage of patients and to devote more attention to patients at risk. In addition, a recent systematic review and critical appraisal indicates that the proposed sophisticated models are frequently poorly reported, with risks of bias, and their reported performance is probably optimistic [16]. For this reason, we opted for the simple model with addition of a sensitivity analysis and another additional classification tree analysis in order to test its robustness. Indeed, internal validation and sensitivity analysis of clinical decision support tool are of prime importance. For binary logistic regression, we performed a sensitivity analysis to test the robustness of Eq 2. This sensitivity analysis revealed that missing a period in the data set, i.e. pre-lockdown (early stage), lockdown (acute stage) and post-lockdown (late stage) have no particular effect rendering the algorithm useful in any stage of the pandemic curve. Despite a reduction of AUC-ROC when removing age, no significant difference was evidenced because of overlapped 95% CIs. However, removing fever alone, both age and fever, both fever and dry cough, and age with fever and dry cough together have a significant effect on decreased performance of Eq 2. Indeed, fever alone or in combination with dry cough and age are the most important significant predictors of the OPS rendering that these demographic characteristic and symptoms especially appealing. The importance of combination of demographic characteristics and symptoms are also evidenced by the use of a matrix of binary Jaccard similarity coefficients. In addition, in this study, complementary clinical decision support tool was used, i.e. a classification tree analysis (CTA). This CTA split the data as explained in materials and methods in order to build the tree with a learning sub-data set and to test the robustness in terms of predictability using another testing sub-data set. This analysis revealed the importance of the same variables identified above as most important predictors (top three), i.e. fever, age in years, and dry cough. The AUC-ROC of this classification tree was similar as the same parameter using Eq 2. The results by two different methods are congruent and robust.

In addition, for qRT-PCR tested patients, the exploration of the trends of Ct values in function of period (pre-lockdown, lockdown and post-lockdown) evidenced a significant decreasing. It should be related to the difference between first occurrence of symptoms and the admission: 3.18 days (S.E.: 2.62 days) in pre-lockdown period, 7.19 days (S.E.: 7.19 days) in lockdown period and 9.22 days (S.E.: 10.73 days) in post-lockdown period. This finding must be in relation with the duration of symptoms of COVID-19. According to ISARIC (2020) [36], this duration is between 1 and 30 days with median = 5 days and IQR = 9 days.

Interesting we found also a significant moderate negative association between the OPS and the Ct values of the qRT-PCR. This finding merits future investigation by means of quantification of viable virus in nasopharyngeal sample of patients with different level of OPS.

Finally, regarding the probability for a patient to become COVID-19 confirmed or unconfirmed by qRT-PCR we proposed, in function of the OPS reached, three levels of operational risk: level 1, high probability for a patient to becomes unconfirmed; level 2, intermediate situation; and level 3, high probability for a patient to become confirmed by qRT-PCR. Those levels should guide clinicians in deciding the appropriate allocation room for patients.

The main limitations of the study include the use of an imperfect test, i.e. with sensibility and specificity less than 100% (qRT-PCR), which was used to discriminate COVID-19 confirmed and unconfirmed cases, and the absence of enrolment of all patients visiting the EDs of Liège University Hospital in the study. Recent reviews recommended the use of nasopharyngeal swab, in place of only nasal or pharyngeal swab, to minimise the false negative patients and indicate that the use of qRT-PCR has a sensitivity decreasing from 90% to 70%, depending on the disease progression but a specificity >99.5% [42, 43]. In addition, recent discovery of some mutation in the E gene of the SARS-CoV-2 can contributed to the loss of sensitivity [44]. Indeed, some errors of patients classification as COVID-19 confirmed or unconfirmed could occur. Therefore, it is recommended to use two gene targets in SARS-CoV-2 qRT-PCR to minimize such rare events.

Another limitation of the study is related to the absence of sufficiently numerous and accurate data on the stratifying by time of presentation of symptoms. Future studies addressing such time stratification would permit the refinement of the current tool.

Finally, regarding the probability for a patient to become COVID-19 confirmed or unconfirmed by qRT-PCR, we proposed, in function of the OPS reached, three levels of operational risk: level 1, high probability for a patient to become unconfirmed; level 2, intermediate situation; and level 3, high probability for a patient to become confirmed by qRT-PCR. Those levels should guide clinicians in deciding the appropriate allocation room for patients. At the hospital, Emergency Departments (EDs) are particularly exposed to the risk of SARS-Cov2 transmission among the different admissions. Differentiating at EDs admission the patients at risk of being confirmed positive for SARS-Cov2 from those who are not is essential to organize an appropriate triage either to COVID area or non-COVID area. The implementation of this tool at an ED nurse triage could permit us to distinguish level 1 patients (low probability of being confirmed positive) requiring an orientation to the non-COVID area and level 3 patients (high probability of being confirmed positive) requiring an orientation to a COVID-designated area. Unfortunately, the score cannot discriminate a certain proportion of patients, which presents intermediate probabilities (level 2). The intermediate category requires the assessment of a medical doctor based on his expertise in the field to direct the patient either to the COVID or non-COVID area. The probability of being confirmed positive between level 2 and 3 ranged from 45% to 55%, respectively. When the probability is higher than 50%, it is needed to promote over-triage instead of under-triage. This was the reason why all patients with a probability over 50% were assigned to the level 3 category.

As currently developed, the OPS is limited to predict the COVID infectivity and is not able to distinguish different levels of severity of disease presentation. In this respect, further development of the OPS should incorporate the severity of COVID-19 disease presentation so as to facilitate more substantiated clinical decisions. However, the severity of disease presentation is also related to the gender (more severe disease presentation were evidenced in males) [45]. Thus, assessment of the severity of disease presentation is a new study as such and it is not the purpose of this study.

## Conclusion

The main interest in the proposed OPS resides in its purely clinical nature for the initial orientation of the patient in a dedicated ward waiting for confirmation by qRT-PCR. By this way, the OPS might be useful at hospital’s admission as a decision support tool for the triage of patients in real-time. Another possibility is to create a pre-hospital triage tool to avoid unnecessary visits to the hospital when testing areas are already overcrowded in peak period. In addition, the introduction of real-time data in an online medical platform could allow for the adaptation of the algorithm of the OPS, rendering its capacity to capture the evolution of clinical patterns over time. This approach could be considered as a strategy to increase resilience against arrival of future emerging infectious diseases, which claims for interdisciplinary and real-time information sharing.

## References

[1] Coronaviridae Study Group of the International Committee on Taxonomy of Viruses (2020). The species severe acute respiratory syndrome-related coronavirus: Classifying 2019-nCoV and naming it SARS-CoV-2. Nature Microbiology, 2020; 5:536–544. 10.1038/s41564-020-0695-z 32123347 PMC7095448

[2] Docherty DM, Rowe SG, Murphy MA, Docherty AB, Harrison EM, Green CA, et al. Features of 20 133 UK patients in hospital with covid-19 using the ISARIC WHO Clinical Characterisation Protocol: prospective observational cohort study. BMJ. 2020; 369:m1985. 10.1136/bmj.m1985 32444460 PMC7243036

[3] Saegerman C, Bianchini J, Renault V, Haddad N, Humblet MF. First expert elicitation of knowledge on drivers of emergence of the COVID-19 in pets. Transbound Emerg Dis. 2020; 10.1111/tbed.13724. 10.1111/tbed.13724 32654387 PMC7405184

[4] Bi Q, Wu Y, Mei S, Ye C, Zou X, Zhang Z, et al. Epidemiology and transmission of COVID-19 in 391 cases and 1286 of their close contacts in Shenzhen, China: A retrospective cohort study. The Lancet Infectious Diseases 2020; S1473–3099(20): 30285–30287. 10.1016/S1473-3099(20)30287-5 32353347 PMC7185944

[5] Burke RM, Midgley CM, Dratch A, Fenstersheib M, Haupt T, Holshue M, et al. Active monitoring of per-sons exposed to patients with confirmed COVID-19—United States, January-February 2020. MMWR. Morbidity and Mortality Weekly Report 2020; 69(9): 245–246. 10.15585/mmwr.mm6909e1 32134909 PMC7367094

[6] Federation of European Heating, Ventilation and Air Conditioning Associations (2020). COVID-19 guidance [Internet]. REHVA. April 3 [cited 2020 May 15]. Retrieved from https://www.rehva.eu/activities/covid-19-guidance.

[7] van Doremalen N, Bushmaker T, Morris DH, Holbrook MG, Gamble A, Williamson BN, et al. Aerosol and Surface Stability of SARS-CoV-2 as Compared with SARS-CoV-1. New England Journal of Medicine 2020; 382(16):1564–1567. 10.1056/NEJMc2004973 PMC7121658 32182409

[8] Zhang R, Li Y, Zhang AL, Wang Y, Molina MJ. Identifying airborne transmission as the dominant route for the spread of COVID-19. Proc Natl Acad Sci USA 2020; 117(26): 14857–14863. 10.1073/pnas.2009637117 32527856 PMC7334447

[9] Wang W, Xu Y, Gao R, Lu R, Han K, Wu G, et al. Detection of SARS-CoV-2 in different types of clinical specimens. JAMA 2020; 323(18):1843–1844. 10.1001/jama.2020.3786 32159775 PMC7066521

[10] Wu Y, Guo C, Tang Y, Qu X, Kuang L, Fang X, et al. Prolonged presence of SARS-CoV-2 viral RNA in faecal samples. Lancet Gastroenterol Hepatol. 2020; 5(5): 434–435. 10.1016/S2468-1253(20)30083-2 32199469 PMC7158584

[11] Wu Z., McGoogan JM. Characteristics of and important lessons from the coronavirus disease 2019 (COVID-19) outbreak in China: Summary of a report of 72 314 caess from the Chinese Center for Disease Control and Prevention. JAMA 2020; 323:1239. 10.1001/jama.2020.2648 32091533

[12] Gilbert A, Brasseur E, Petit M, Donneau A-F, Nguyet Diep A, Hetzel Campbell S, et al. Immersion in an emergency department triage center during the COVID-19 outbreak: first report of the Liège University Hospital experience. Acta Clin Belg. 2020;1–7. 10.1080/17843286.2020.1778348 32531181

[13] Iserson KV, Moskop JC. Triage in medicine, Part I: concept, history, and types. Ann Emerg Med. 2007; 49(3):275–281. 10.1016/j.annemergmed.2006.05.019 17141139

[14] Struyf T, Deeks JJ, Dinnes J, Takwoingi Y, Davenport C, Leeflang M, et al. Signs and symptoms to determine if a patient presenting in primary care or hospital outpatient settings has COVID-19 disease. Cochrane Database Syst Rev. 2020; 7(7):CD013665. 10.1002/14651858.CD013665 32633856 PMC7386785

[15] McRae MP, Dapkins IP, Sharif I, Anderman J, Fenyo D, Sinokrot O, et al. Managing COVID-19 With a Clinical Decision Support Tool in a Community Health Network: Algorithm Development and Validation. J Med Internet Res. 2020;22(8):e22033. 10.2196/22033 32750010 PMC7446714

[16] Wynants L, Van Calster B, Collins GS, Riley RD, Heinze G, Schuit E, et al. Prediction models for diagnosis and prognosis of covid-19 infection: systematic review and critical appraisal. BMJ. 2020;369:m1328. 10.1136/bmj.m1328 32265220 PMC7222643

[17] Guan WJ, Ni ZY, Hu Y, Liang WH, Ou CQ, He JX, et al. Clinical Characteristics of Coronavirus Disease 2019 in China. N Engl J Med. 2020; 382(18):1708–1720. 10.1056/NEJMoa2002032 32109013 PMC7092819

[18] Sciensano (2020a). Définition de cas, indications de demande d’un test et déclaration obligatoire de cas COVID-19. Sciensano, Bruxelles, Belgique. 4 pages. https://covid-19.sciensano.be/sites/default/files/Covid19/COVID-19_Case%20definition_Testing_FR.pdf

[19] Lechien JR, Chiesa-Estomba CM, Place S, Van Laethem Y, Cabaraux P, Mat Q, et al. Clinical and epidemiological characteristics of 1420 European patients with mild-to-moderate coronavirus disease 2019. J Intern Med. 2020; 288(3): 335–344. 10.1111/joim.13089 32352202 PMC7267446

[20] Carfì A, Bernabei R, Landi F, Gemelli Against COVID-19 Post-Acute Care Study Group. Persistent Symptoms in Patients After Acute COVID-19. JAMA 2020; 324(6):603–605. 10.1001/jama.2020.12603 32644129 PMC7349096

[21] Hausmann JS, Berna R, Gujral N, Ayubi S Hawkins J, Brownstein JS, et al. Using Smartphone Crowdsourcing to Redefine Normal and Febrile Temperatures in Adults: Results from the Feverprints Study. J Gen Intern Med. 2018; 33(12):2046–2047. 10.1007/s11606-018-4610-8 30105481 PMC6258625

[22] Corman VM, Landt O, Kaiser M, Molenkamp R, Meijer A, Chu DK, et al. Detection of 2019 novel coronavirus (2019-nCoV) by real-time RT-PCR. Euro Surveill. 2020; 25(3):pii = 2000045. 10.2807/1560-7917.ES.2020.25.3.2000045 31992387 PMC6988269

[23] Petrie A, Watson P. (2013). Statistics for veterinary and animal science. This edition, John Wiley & Sons, Ltd, West Sussex, UK, 391 pages.

[24] Saegerman C, Speybroeck N, Dal Pozzo F, Czaplicki G. Clinical indicators of exposure to *Coxiella burnetii* in dairy herds. Transbound Emerg Dis. 2015; 62(1):46–54. 10.1111/tbed.12070 23480126

[25] Chung NC, Miasojedow B, Startek M, Gambin A. Jaccard/Tanimoto similarity test and estimation methods for biological presence-absence data. BMC Bioinformatics 2019; 20(Suppl 15):644. 10.1186/s12859-019-3118-5 31874610 PMC6929325

[26] Preux PM, Odermatt P, Perna A, Marin B, Vergnenégre A. Qu’est-ce qu’une régression logistique? Rev. Mal. Respir. 2005; 22:159–162. 10.1016/s0761-8425(05)85450-6 15968772 PMC7134681

[27] Breiman I, Friedman JH, Olsen RA, Stone CJ. (1984). Classification and Regression Trees. Wadsworth International Group Belmont, Monterey, CA, USA.

[28] Saegerman C., Speybroeck N., Roels S., Vanopdenbosch E., Thiry E., Berkvens D. (2004). Decision support tools for clinical diagnosis of disease in cows with suspected bovine spongiform encephalopathy. J Clin Microbiol., 42(1), 172–178. 10.1128/jcm.42.1.172-178.2004 14715749 PMC321688

[29] Saegerman C., Porter S.R., Humblet M.F. (2011). The use of modelling to evaluate and adapt strategies for animal disease control. Rev Sci Tech. 2011, 30(2), 555–569. 10.20506/rst.30.2.2048 21961226

[30] Speybroeck N, Berkvens D, Mfoukou-Ntsakala A, Aerts M, Hens N, Van Huylenbroeck G, et al. Classification trees versus multinomial models in the analysis of urban farming systems in Central Africa. Agric. Syst. 2004; 80:133–149.

[31] Steinberg D, Colla P. (1997). CART-Classification and Regression Trees. Instruction manual, Salford Systems, San Diego, CA.

[32] Nations United. (1982). Provisional guidelines on standard international age classifications. United Nations, Department of international economic and social affairs, New York, NY, USA. Document ST/ESA/STAT/SEA.M/74, 28 pages. https://unstats.un.org/unsd/publication/SeriesM/SeriesM_74e.pdf.

[33] Iaccarino G, Grassi G, Borghi C, Carugo S, Fallo F, Ferri C, et al. Gender differences in predictors of intensive care units admission among COVID-19 patients: The results of the SARS-RAS study of the Italian Society of Hypertension. PLoS One 2020: 15(10):e0237297. 10.1371/journal.pone.0237297 33022004 PMC7537902

[34] Sun Y, Koh V, Marimuthu K, Tek Ng O, Young B, Vasoo S, et al. Epidemiological and Clinical Predictors of COVID-19. Clin Infect Dis. 2020; 71(15):786–792. 10.1093/cid/ciaa322 32211755 PMC7542554

[35] Ibrahim Olayinka Rasheed, Suleiman Bello Muhammed, Abdullahi Suleiman Bello, Oloyede Taofeek, Sanda Abdallah, Gbadamosi Maruf Sanusi, et al. Epidemiology of COVID-19 and Predictors of Outcome in Nigeria: A Single-Center Study. Am J Trop Med Hyg. 2020; in press. 10.4269/ajtmh.20-0759 33124545 PMC7695094

[36] ISARIC, International Severe Acute Respiratory and Emerging Infections Consortium (2020). COVID-19 Report: 19 May 2020 [Internet]: ISARIC; 2020. Available from: https://media.tghn.org/medialibrary/2020/05/ISARIC_Data_Platform_COVID-19_Report_19MAY20.pdf.

[37] Swets J. Measuring the accuracy of diagnostic systems. Science 1988; 240:1285–1293. 10.1126/science.3287615 3287615

[38] Porter RS, Leblond A, Lecollinet S, Tritz P, Cantile C, Kutasi O, et al. (2011). Clinical diagnosis of West Nile Fever in Equids by classification and regression tree (CART) analysis and comparative study of clinical appearance in three European countries. Transbound Emerg Dis., 58(3), 197–205. 10.1111/j.1865-1682.2010.01196.x 21208395

[39] Saegerman C., Humblet M.F., Porter S.R., Zanella G., Martinelle L. (2012). Evidence-based early clinical detection of emerging diseases in food animals and zoonoses: two cases. Vet Clin North Am Food Anim Pract., 28(1), 121–131. 10.1016/j.cvfa.2012.01.001 22374122

[40] Zanella G, Martinelle L, Guyot H, Mauroy A, De Clercq K, Saegerman C. Clinical pattern characterization of cattle naturally infected by BTV-8. Transbound Emerg Dis. 2013; 60(3):231–237. 10.1111/j.1865-1682.2012.01334.x 22571462

[41] Menni C, Valdes AM, Freidin MB, Sudre CH, Nguyen LH, Drew DA, et al. Real-time tracking of self-reported symptoms to predict potential COVID-19. Nat Med. 2020; 26(7):1037–1040. 10.1038/s41591-020-0916-2 32393804 PMC7751267

[42] Sciensano (2020b). Fact sheet COVID-19 disease (SARS-CoV-2 virus). Version updated at September 21, 2020. Sciensano, Bruxelles, Belgium, 48 pages. https://covid-19.sciensano.be/sites/default/files/Covid19/COVID-19_fact_sheet_ENG.pdf

[43] Giri B, Pandey S, Pokharel K, Ligler FS, Neupane BB. Review of analytical performance of COVID-19 detection methods. Analytical and Bioanalytical Chemistry, 2020; in press. 10.1007/s00216-020-02889-x 32944809 PMC7498299

[44] Artesi M, Bontems S, Göbbels P, Franckh M, Maes P, Boreux R, et al. A Recurrent Mutation at Position 26340 of SARS-CoV-2 Is Associated with Failure of the E Gene Quantitative Reverse Transcription-PCR Utilized in a Commercial Dual-Target Diagnostic Assay. J Clin Microbiol. 2020; 58(10):e01598–20. 10.1128/JCM.01598-20 32690547 PMC7512182

[45] Ueyama H, Kuno T, Takagi H, Krishnamoorthy P, Vengrenyuk Y, Sharma SK, et al. Gender Difference Is Associated With Severity of Coronavirus Disease 2019 Infection: An Insight From a Meta-Analysis. Crit Care Explor. 2020; 2(6):e0148. 10.1097/CCE.0000000000000148 32696011 PMC7314340

